# The implications of climatic changes on food and water-borne diseases in Malaysia: a case study of Kelantan, Terengganu, Johor and Melaka

**DOI:** 10.1186/1471-2458-14-S1-P22

**Published:** 2014-01-29

**Authors:** Noor Artika Hassan, Jamal Hisham Hashim, Zubaidi Johar, Mohd Syazwan Faisal

**Affiliations:** 1 United Nations University-International Institute for Global Health, UKM Medical Centre, Jalan Yaacob Latif , Bandar Tun Razak , 56000 Kuala Lumpur, Malaysia; 2 National Hydraulic Research Institute of Malaysia (NAHRIM), Ministry of Natural Resources & Environment (NRE), 43300 Seri Kembangan Selangor Darul Ehsan, Malaysia

## Background

Climate change has been recognised as the most pressing environmental problem humans will face in the 21st century. Intergovernmental Panel on Climate Change (IPCC) estimates that the global mean surface temperature has increased 0.74ºC between 1905 and 2005, and predicts an increase of 2 to 4.5ºC over the next 100 years. In Malaysia, observed surface temperature data for the last four decades estimates an increase of between 2.7 to 4.0ºC per century. Climate change is suspected to have adverse human health impacts. This study is an attempt to quantify climate-induced increases in morbidity rates associated with food and water-borne diseases.

## Materials and methods

Monthly cases of food and water related diseases data (food poisoning, leptospirosis, cholera, hepatitis A, typhoid, and dysentery) between the year 2000 and 2012 will be obtained from the Ministry of Health Malaysia. Climate data, including monthly average temperature and rainfall, will be obtained from the Malaysian Meteorological Department and National Hydraulic Research Institute of Malaysia. Population projections in Malaysia, up to the year 2100 will be adopted from the World Population Prospects (UN, 2010). Climate projections will be forecasted by the Hadley Centre of UK Meteorological Office’s RCM known as PRECIS to predict the increase in monthly temperature until 2070-2100 under the A1B scenario. The projection will be downscaled to a 5 km by 5 km grid resolution. A Poisson generalized linear model will be developed to quantify the relationship between climatic parameters and the number of reported food and water-borne disease cases.

## Results

Altered weather patterns and changes in precipitation and temperature resulting from climate change are likely to affect the distribution and incidence of food and water-borne diseases in Malaysia.

## Conclusions

Information regarding the impact of climate change on food and waterborne diseases is very limited. Therefore, findings from this study will be beneficial for the policy makers for an adaptive strategy to enhance health systems in Malaysia and to improve on water resources planning and management.

